# Editors’ page

**DOI:** 10.1016/j.ajpc.2020.100135

**Published:** 2020-12-01

**Authors:** Sergio Fazio, Nathan D. Wong

This fourth issue of the *Journal* completes an overwhelmingly successful launch year and sets our trajectory straight toward the calmer waters enjoyed by more established publications, with a steady equilibrium of the right type of papers in different stages of processing. As predicted, the *Journal* is indeed filling an important gap in the scientific literature by providing a forum for practitioners and translational researchers in the wide area of preventive cardiology to feature work ranging from state-of-the-art reviews to practice recommendations, from teaching and training to pragmatic research. We are approaching the 100 mark on submissions, representing not only the diversity of specialists in preventive cardiology – physicians, pharmacists, dietitians, and researchers - but also the global reach of our discipline, with contributions from several countries, including Turkey, India, Australia, and Argentina. The *Journal* could not have gotten to this sustainable point so fast without the tremendous support from the American Society for Preventive Cardiology, our expert colleagues at Elsevier Publishing, the diverse international editorial board, and in particular our tight team of associate editors.

We are pleased to present to you this year’s final issue of the *American Journal of Preventive Cardiology*, on schedule and on target with more than a dozen contributions, including several reviews on, among other issues, the European and US cholesterol guidelines, the burden of cardiometabolic risk in the Asia-Pacific region, nutritional strategies for cardiovascular disease prevention, and the hot topic of inflammation in cardiovascular disease. Some excellent original contributions are included, touching on issues ranging from racial disparities in cardiovascular disease to hospitalization rates in persons with diabetes to the cardio-metabolic risk in those with non-alcoholic fatty liver disease.

We are working hard at making 2021 a boom year for the *Journal*, and the large and growing number of quality submissions currently in process promises to make the *AJPC* the “to go” place for implementable work in preventive cardiology. We are now in the process of applying for PubMed indexing, as testimony to the incredible progress achieved in just one year.

It is the hope of the *Journal* to continue its outreach strategy to those engaged in the practice of preventive cardiology around the world, encouraging submissions featuring practice models, training systems, pragmatic research, and practice recommendations representing our field at an international level. Further, the *Journal* will continue to be a key educational resource for the members of the American Society for Preventive Cardiology, and a forum to feature their research and perspectives on the practice of this medical art we call Preventive Cardiology.

We count on the continued support of our editorial board and of experts in the field from around the world in reviewing and submitting work and in helping us establish AJPC as the top target publication for those who live and breathe preventive cardiology. With all challenges that 2020 has thrown into our professional and daily lives, we must celebrate the small victories and everything that brings us together, and look into the future with love, confidence, and passion. Our best wishes of a healthy 2021 to our *Journal* and to all in this beautiful world of ours.

